# Supplementary material for: Zebrafish model reveals developmental and hematopoietic functions of ADAMTS13
Source: Biol Open. 2025 Oct 24;14(10):bio062265. doi: 10.1242/bio.062265 (PMC12584400; doi:10.1242/bio.062265)
Supplement: Supplementary information [file biolopen-14-062265-s1.pdf]

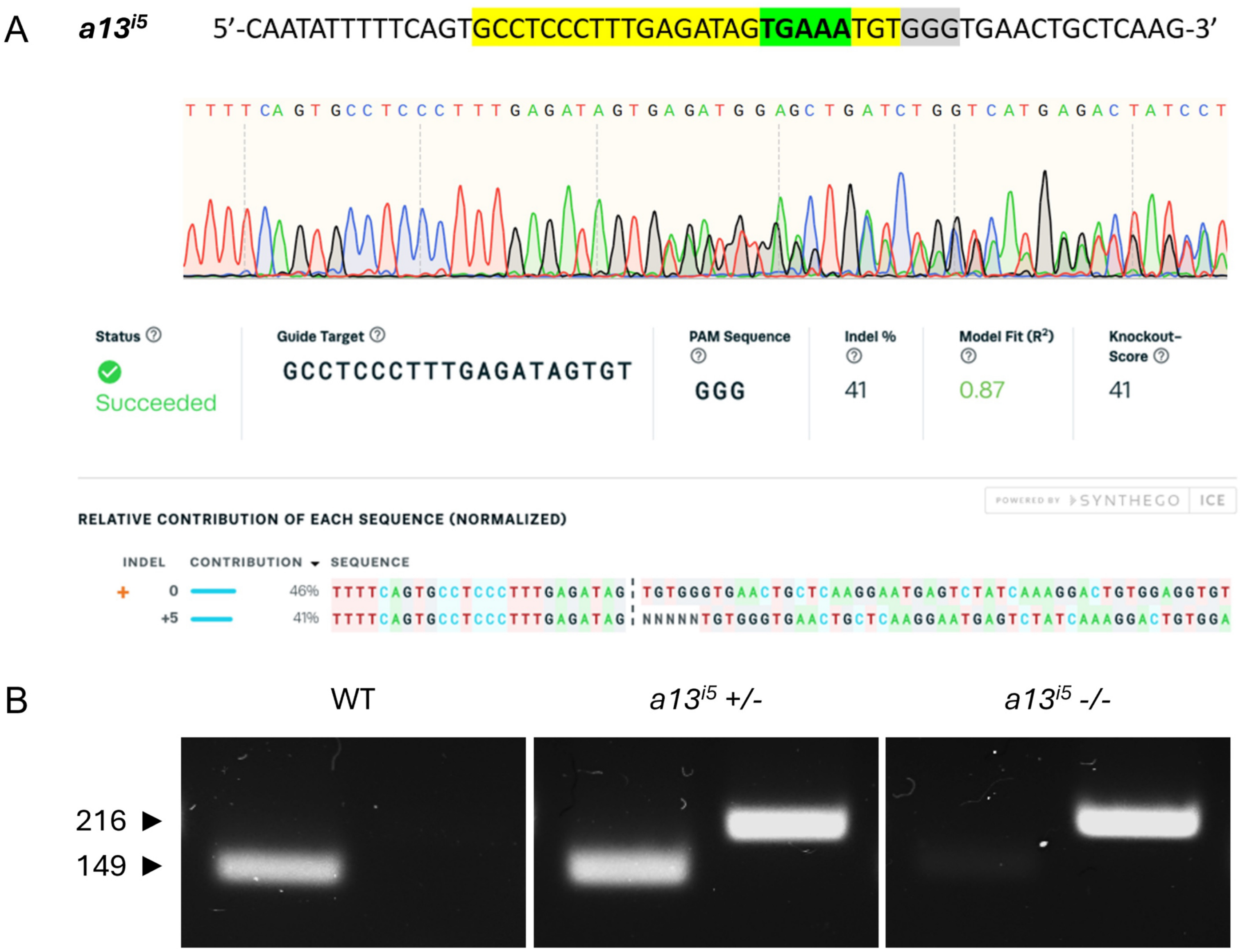

Fig. S1. *a13^{i5}* fish carry a frameshift-causing 5 bp insertion: (A) Sanger sequencing of *a13^{i5}* heterozygous fish (upper panel) and analysis through Synthego ICE tool (lower panel). (B) Genotyping through agarose gel electrophoresis using ASOs. Yellow: sgRNA. Grey: PAM sequence. Green: 5 bp insertion.

Biology Open • Supplementary information

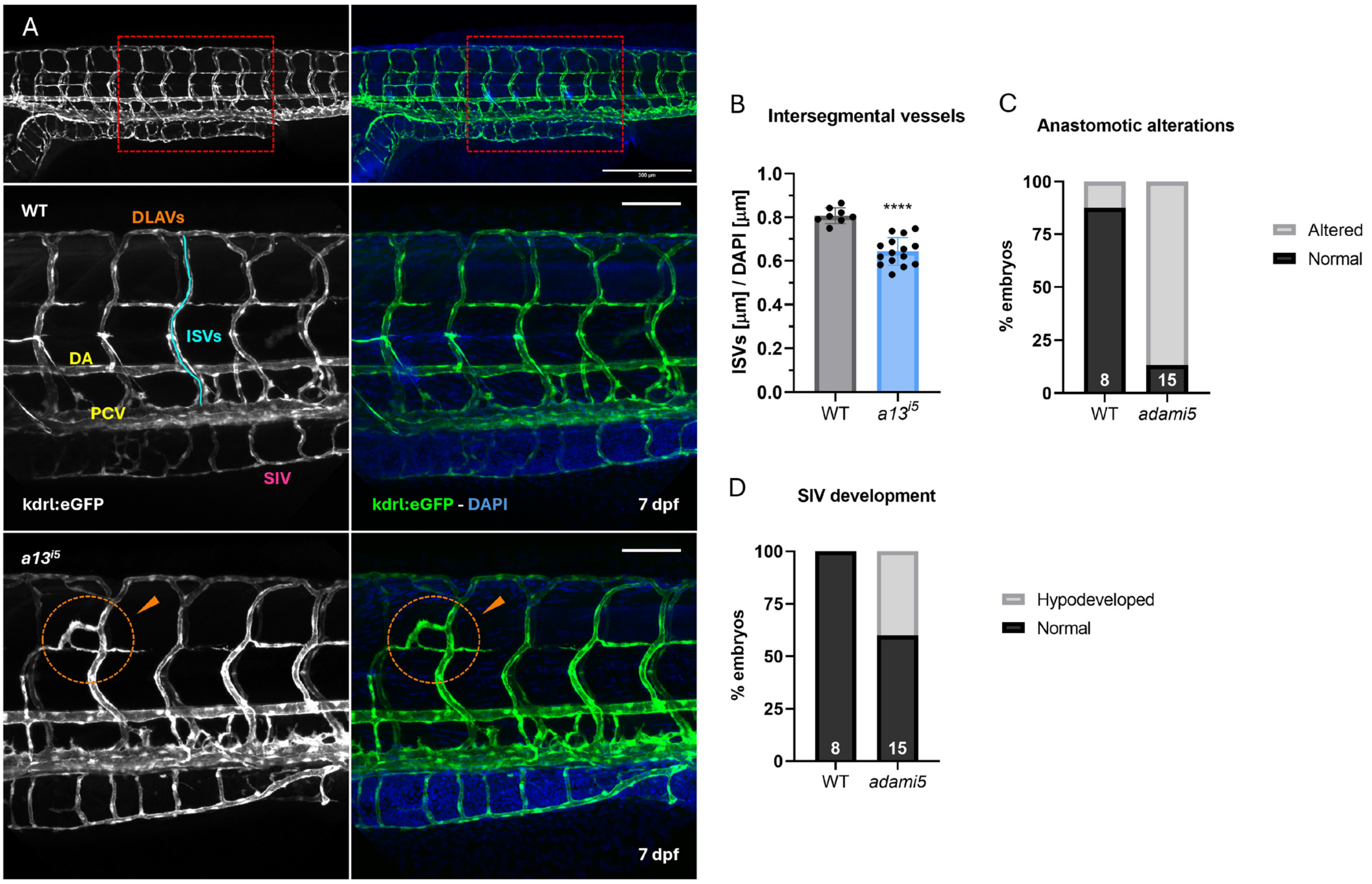

Fig. S2. ADAMTS13 deficiency impairs SIV development in zebrafish: (A) representative confocal images of the trunk vasculature of *casper;kdrl:eGFP* transgenic embryos at 7 dpf. Top panels show an overview of the tail vasculature (10X), the red rectangle highlights the area of the trunk considered for the analysis. Scale bar: 300 µm. Mid panels show a WT embryo, *kdrl:eGFP* in grey (left), and the merge with DAPI (right). Lower panels show an *a13*^{i5} mutant embryo, *kdrl:eGFP* in grey (left) and the merge with DAPI (right). Scale bar: 100 µm. (B) Quantification of ISV length [µm] normalized to DAPI in WT (grey) and *a13*^{i5} (blue) embryos. (C) Percentage of embryos with normal (black) or altered (grey) anastomotic connections in WT and *a13*^{i5} mutants. (D) Percentage of embryos with normal (black) or hypodeveloped (grey) SIV plexus in WT and *a13*^{i5} mutants. Data are shown as mean ± SD. Each dot represents a biological replicate, **** $p \leq 0.0001$ by unpaired Student's t-test. DA: dorsal aorta, DLAV: dorsal longitudinal anastomotic vessel, ISV: intersegmental vessel, PCV: posterior cardinal vein, SIV: subintestinal vein.

Table S1. PCR primers.

Forward: 5'-CACAAATGAGGAGTCGGGCT-3'

Reverse: 5'-TCAGCTGCTCGCAACACATA-3'

RT-qPCR primers

gata1	forward	5'-TGAATGTGTGAATTGTGGTG-3'
	reverse	5'-ATTGCGTCTCCATAGTGTTG-3'
il-1β	forward	5'-TTTGTGGGAGACAGACGGTG-3'
	reverse	5'-TCAGGGCGATGATGACGTTC-3'
il-6	forward	5'-TCAACTTCTCCAGCGTGATG-3'
	reverse	5'-TCTTTCCCTCTTTTCCTCCTG-3'
marco	forward	5'-GACAGACAGGAGATCCTGG-3'
	reverse	5'-CTCCACGCTCACCTTTGAGAC-3'
mpeg1	forward	5'-CATGTCGTGGCTGGAACAGA-3'
	reverse	5'-ATGGTTACGGACTTGAACCCG-3'
mpx	forward	5'-TGATGTTTGGTTAGGAGGTG-3'
	reverse	5'-GAGCTGTTTTCTGTTTGGTG-3'
tnfα	forward	5'-GGCCTTTTCTTCAGGTGGCT-3'
	reverse	5'-AGTGGCTTTTTGCCTCCGTA-3'
vegfa	forward	5'-AGAAAGAAAACCACTGTGAG-3'
	reverse	5'-AGGAATGTTCTTCCTTAGGT-3'
ube2a	forward	5'-TGACTGTTGACCCACCTTACAG-3'
	reverse	5'-CAAATAAAAGCAAGTAACCCC-3'

Biology Open • Supplementary information

T7 WISH PCR primers.

Forward	5’-GAAAAACGTGTTCTGGCTCT-3’
Reverse	5’-TAATACGACTCACTATAGGG)GACCCCATTCGCAAACACT-3’

Genotyping ASOs

wild-type	forward	5’-GGGTTAAGGTTTACAGAAAAGCCATTA-3’
	reverse	5’-CCTTGAGCAGTTCACCCACACTAT-3’
adamts13^{i5}	forward	5’-GCCTCCCTTTGAGATAGTGAAATG-3’
	reverse	5’-CAGCTGCTCGCAACACATATCC-3’

Biology Open • Supplementary information

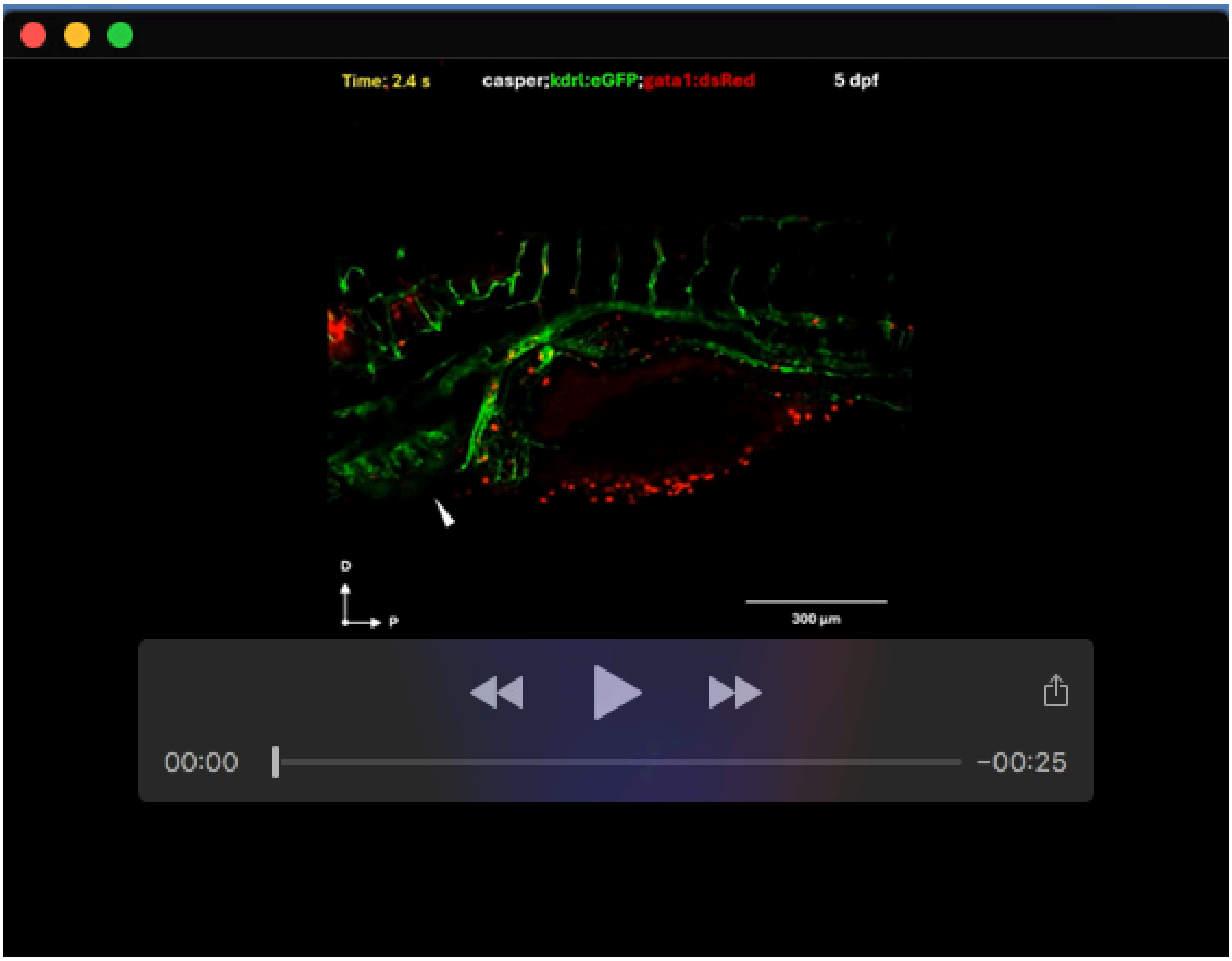

Movie 1. representative confocal timelapse focused on the heart of *casper;kdrl:eGFP;gata1:dsRed* transgenic zebrafish at 5 dpf. After tail excision, the clot seals the wound and blocks blood circulation at the site of the cut. The white arrow shows that the heart is still beating after this process. 10X dry objective. Scale bar: 300 µm.
